# A53T-α-synuclein overexpression in murine locus coeruleus induces Parkinson’s disease-like pathology in neurons and glia

**DOI:** 10.1186/s40478-018-0541-1

**Published:** 2018-05-10

**Authors:** Martin Timo Henrich, Fanni Fruzsina Geibl, Bolam Lee, Wei-Hua Chiu, James Benjamin Koprich, Jonathan Michael Brotchie, Lars Timmermann, Niels Decher, Lina Anita Matschke, Wolfgang Hermann Oertel

**Affiliations:** 1 Department of Neurology, Philipps University Marburg, Baldingerstraße 1, 35043 Marburg, Germany; 2 Krembil Research Institute, Toronto Western Hospital, University Health Network, Toronto, ON Canada; 3 Department of Physiology and Pathophysiology, Philipps University Marburg, 35043 Marburg, Germany

**Keywords:** Parkinson’s disease, Locus coeruleus, Alpha-synuclein, Adeno-associated viral vectors, Prodromal mouse model, Microglia, noradrenergic neurons

## Abstract

Degeneration of noradrenergic locus coeruleus neurons occurs during the prodromal phase of Parkinson’s disease and contributes to a variety of non-motor symptoms, e.g. depression, anxiety and REM sleep behavior disorder. This study was designed to establish the first locus coeruleus α-synucleinopathy mouse model, which should provide sufficient information about the time-course of noradrenergic neurodegeneration, replicate cardinal histopathological features of the human Parkinson’s disease neuropathology and finally lead to robust histological markers, which are sufficient to assess the pathological changes in a quantitative and qualitative way. We show that targeted viral vector-mediated overexpression of human mutant A53T-α-synuclein in vivo in locus coeruleus neurons of wild-type mice resulted in progressive noradrenergic neurodegeneration over a time frame of 9 weeks. Observed neuronal cell loss was accompanied by progressive α-synuclein phosphorylation, formation of proteinase K-resistant α-synuclein-aggregates, accumulation of Ubi-1- and p62-positive inclusions in microglia and induction of progressive micro- and astrogliosis. Apart from this local pathology, abundant α-synuclein-positive axons were found in locus coeruleus output regions, indicating rapid anterograde axonal transport of A53T-α-synuclein. Taken together, we present the first model of α-synucleinopathy in the murine locus coeruleus, replicating essential morphological features of human Parkinson’s disease pathology. This new model may contribute to the research on prodromal Parkinson’s disease, in respect to pathophysiology and the development of disease-modifying therapy.

## Introduction

Parkinson’s disease (PD) is the second most common neurodegenerative disorder [2] characterized by progressive degeneration of dopaminergic (DA) substantia nigra (SN) neurons and their striatal axon terminals [40, 68]. One characteristic neuropathological hallmark of PD are intracytoplasmic eosinophilic inclusions, the so-called Lewy bodies, which develop in specific brain regions in a spatio-temporal pattern and consist predominantly of misfolded α-synuclein (aSYN) [11, 83]. The finding that duplications, triplications or missense mutations (e.g. A53T, A30P or G46 L) of the aSYN gene (SNCA) cause familial forms of PD [46, 67] has justified the assumption that aSYN plays a crucial role in the pathogenesis of PD.

Only within the last 20 years it is accepted that PD cannot solely be understood as a disease associated with the degeneration of DA SN neurons, as the PD pathology involves the central, peripheral, autonomic and enteric nervous system [11, 12, 16, 79, 95]. The degeneration of DA SN neurons and the onset of motor dysfunction are preceded by a latency of several years, if not decades, in which the PD pathology develops in brain regions outside the DA SN. This phase, termed prodromal PD, is clinically characterized by the occurrence of certain non-motor symptoms, e.g. hyposmia, constipation, depression and idiopathic REM sleep behavior disorder [31, 53, 84]. Since the prodromal phase is seen as the ideal time window for applying disease-modifying therapy [60, 92], it is of high importance to establish animal models, which allow testing of new and future therapeutic approaches on brain structures that are affected during prodromal PD. The noradrenergic locus coeruleus (LC), a monoaminergic nucleus located in the pontine brainstem [3, 8], plays a crucial role during the prodromal phase of PD and represents therefore an ideal brain structure for such in-depth characterization in an experimental animal model [97]. Dysfunction and degeneration of neurons in the LC region are associated with several of the above listed non-motor symptoms, including depression, signs of reduced arousal, anxiety and REM sleep behavior disorder (RBD) [17, 27, 93]. Neuropathological analysis of human PD brain samples revealed up to 80% LC neuronal cell loss in PD patients, thereby exceeding the degree of SN neurodegeneration in the same individuals [58, 99]. Moreover, experimental evidence indicates that toxin-induced LC cell loss sensitized DA SN neurons for neurodegeneration [9, 22], whereas noradrenergic hyperinnervation resulted in neuroprotective effects [41]. This data implies that LC neurodegeneration itself plays a double role by firstly being responsible for several non-motor symptoms and secondly for accelerating the progression of PD at the nigral level [27]. LC cells exhibit a common at-risk phenotype compared to other neuronal populations such as the DA nigral neurons and the cholinergic neurons of the dorsal motor nucleus of the vagal nerve which undergo neurodegeneration in PD [6, 78]. LC neurons integrate information from a broad range of different brain regions and broadcast information with extensively branched and thinly myelinated axons throughout the complete neuroaxis [8, 75]. Furthermore, they exhibit an intrinsic pacemaking activity, generating action potentials continuously [55] thereby raising their basal metabolic stress level [78].

In this study, we have characterized the first model of α-synucleinopathy in the murine LC. We show that targeted viral vector-mediated overexpression of human mutant A53T-aSYN in vivo in LC neurons of wild-type mice resulted in progressive LC neurodegeneration over a time frame of 9 weeks. Observed LC cell loss was accompanied by prominent and over time increasing micro- and astrogliosis. In addition, our data revealed accumulation of phosphorylated aSYN, progressive aggregation of aSYN as demonstrated by proteinase K-resistant aSYN aggregates and Ubi-1- and p62-positive inclusions comparable with findings from human PD samples. Co-staining with different cellular markers revealed that the p62- and Ubi-1-positive aggregates were found exclusively in microglial cells, while being absent in neurons, astrocytes and oligodendrocytes. Beside this local LC pathology, we observed abundant aSYN-positive axons in a high number of LC output regions, indicating rapid anterograde axonal transport of the human aSYN. In conclusion, our new murine LC model replicated cardinal morphological features of human PD pathology.

## Materials and methods

### Animals

A total of 70 wild-type male C57BL/6 N mice (Charles River, Sulzfeld, Germany), 8 weeks old at the beginning of the experiment, were used. Mice were housed in individually ventilated cages with ad libitum access to food and water under a 12 h/12 h light-dark cycle. All procedures performed in studies involving animals were in accordance with the ethical standards of the institution at which the studies were conducted (Regierungspräsidium Giessen, Germany V54–19 c 20 15 h 01 MR 20/15 Nr. 66/2015).

### Recombinant adeno-associated viral (rAAV) vectors and stereotactic injection

Two different recombinant adeno-associated viral (rAAV) vectors of a mixed 1/2 serotype were used to overexpress human mutant-A53T-aSYN (rAAV1/2-CMV/CBA-human-A53T-aSYN-WPRE-BGH-pA (rAAV1/2-A53T-aSYN); viral titer 5.1 × 10^12^ gp/ml, purchased from GeneDetect) or luciferase (rAAV1/2-CMV/CBA-luciferase-WPRE-BGH-pA (rAAV1/2-Luc), viral titer 5.0 × 10^12^ gp/ml, purchased from GeneDetect). Each of the two vectors was driven by a chicken beta actin (CBA) promoter combined with a cytomegalovirus (CMV) immediate early enhancer sequence and a woodchuck post-transcriptional regulatory element (WPRE) to assess a high transcription rate [38, 44]. For stereotactic delivery of the rAAV vectors, mice were anesthetized with 100 mg/kg ketamine and 5 mg/kg xylazine via intraperitoneal injection. A volume of 1.25 μl of rAAV1/2-A53T-aSYN or rAAV1/2-Luc was stereotactically injected in the right LC region using a microinjector (UltraMicro Pump UMP3, World Precision Instruments) with a velocity of 125 nl/min based on the following coordinates: ML − 0.9 mm, AP -5.4 mm and DV -3.65 mm relative to Bregma [66].

### Tissue preparation

Mice were sacrificed through transcardial perfusion with 0.1 M phosphate-buffered saline (PBS) for 5 min followed by 4% ice-cold paraformaldehyde (PFA) in 0.1 M phosphate buffer (PB) (pH 7.4) for 5 min using a supply pump at a rate of 10 ml/min. Brains were carefully removed and post-fixed in 4% PFA for 3 days and then transferred to 30% sucrose solution for 3 days for cryoprotection. Brains were cut into 20 μm thick coronal sections using a cryostat microtome (Leica CM3050 S, Nussloch, Germany). Sections were then stored at 4 °C in cryoprotect-solution (1:1:3 volume ratio of ethylenglycol, glycerol and 0.1 M PB) until further processing.

### Immunohistochemistry with 3,3-diaminobenzidine (DAB)

Free-floating sections containing the LC/SN region were washed in 0.1 M PB and quenched with 3% H_2_O_2_ and 10% methanol for 15 min. After a second wash, sections were blocked in 5% normal donkey serum with 0.3% Triton X-100 in 0.1 M PB for 1 h before incubating them overnight with primary antibodies against TH, p-aSYN, Ubi-1 or p62 (Table 1) at 4 °C in the same blocking solution. On the second day, sections were washed in 0.1 M PB for 20 min and then incubated with the appropriate biotinylated secondary antibody (Table 1) for 1 h, followed by incubation in avidin-biotin-peroxidase solution (ABC Elite, Vector Laboratories) for 1 h before initiating the color reaction with 5% DAB (Serva), diluted in 0.1 M PB with 0.02% H_2_O_2_. All DAB-stained sections were mounted, dried, counterstained with cresyl-violet and coverslipped with mounting gel (Corbit-Balsam, Eukitt). Brightfield images were acquired using an AxioImager M2 microscope (Zeiss) equipped with an Axiocam 506 color camera (Zeiss).

**Table 1:** Characteristics of the primary and secondary antibodies

Antigen	Host	Cat. No.	Manufacturer	Dilution
Tyrosine Hydroxylase	Rabbit	AB152	Merck Millipore	1:1000
Tyrosine Hydroxylase	Sheep	AB1542	Merck Millipore	1:1000
AAV VP1/VP2/VP3	Rabbit	61,084	Progen	1:250
Alpha-synuclein (p-S129)	Rabbit	ab51253	Abcam	1:2000
Alpha-synuclein (syn211)	Mouse	AHB0261	ThermoFisher	1:1000
Luciferase	Goat	NB100–1677	Novus Biologicals	1:250
GFAP	Chicken	ab4674	Abcam	1:2000
IbA1	Rabbit	019–19,741	Wako	1:500
Ubiquitin (Ubi-1)	Mouse	ab7254	Abcam	1:2000
SQSTM1/p62	Mouse	ab56416	Abcam	1:2000
Olig2	Rabbit	ab109186	Abcam	1:500
MAP2	Chicken	ab5392	Abcam	1:2000
Anti-rabbit AlexaFluor488	Donkey	A-21206	Invitrogen	1:1000
Anti-goat AlexaFluor488	Donkey	A-11055	Invitrogen	1:1000
Anti-mouse AlexaFluor488	Donkey	A-2102	Invitrogen	1:1000
Anti-chicken Cy3	Donkey	703–165-155	Jackson ImmunoResearch	1:1000
Anti-mouse Cy3	Donkey	715–165-150	Jackson ImmunoResearch	1:1000
Anti-goat Cy3	Donkey	705–165-147	Jackson ImmunoResearch	1:1000
Biotinylated anti-rabbit	Donkey	711–065-152	Jackson ImmunoResearch	1:1000
Biotinylated anti-mouse	Donkey	715–065-151	Jackson ImmunoResearch	1:1000
Biotinylated anti-goat	Donkey	705–065-147	Jackson ImmunoResearch	1:1000
Streptavidin AlexaFluor647	Donkey	016–600-084	Jackson ImmunoResearch	1:1000

### Immunofluorescence staining

Sections were washed in 0.1 M PB, then blocked in 10% normal donkey serum with 0.3% Triton X-100 in 0.1 M PB for 1 h before incubating them with primary antibodies (Table 1) at 4 °C in the same blocking solution overnight. On the second day, sections were washed in 0.1 M PB containing 0.3% Triton X-100 and then incubated with fluorophore-conjugated, species-specific secondary antibodies (Table 1) for 2 h at room temperature in 0.1 M PB containing 0.3% Triton X-100 and 10% normal donkey serum. Before mounting sections were washed for 25 min in 0.1 M PB containing 0.3% Triton X-100. Exceptions from this general protocol were made for staining luciferase, p-aSYN, IbA1 and Olig2, where after primary antibody incubation a biotinylated species-specific secondary antibody was used to further improve signal to noise by conjugation with streptavidin. Images were acquired using an AxioImager M2 microscope (Zeiss) equipped with an ORCA-Flash4.0 LT CMOS camera (Hamamatsu C11440-42 U). For confocal images, a TCS SP8 microscope (Leica) was used. Images were processed with FIJI image software [81] to enhance signal-to-noise. Image data for 3D reconstructions were obtained with a Zeiss Spinning Disc Microscope (Axio Observer Z1) equipped with an Axiocam MRm (Zeiss) and an Evolve 512 EMCCD Camera (Photometrics) and post-processed with ZEN 2012 software (Zeiss).

### Proteinase K treatment

To analyze the formation of insoluble aggregates, sections were digested with Proteinase K (PK) using a modified protocol described elsewhere [21, 86]. 20 μm thick sections with 120 μm interslice distance containing the complete LC region were washed in 0.1 M PB and subsequently digested in 0.1 M PB containing 0.3% Triton X-100 and 12 μg/ml PK (Cat. No. 4333793, Invitrogen) at 65 °C for 10 min. To visualize insoluble aggregates, digested sections were double stained against human aSYN, p62, Ubi-1 or luciferase in combination with TH (Table 1), following the fluorescence staining protocol described above. Complete absence of TH immunoreactivity served as an indicator for successful PK digestion, thus sections in which TH immunoreactivity was still visible were excluded from analysis implicating an incomplete protein digestion. Images were acquired using an AxioImager M2 microscope (Zeiss) equipped with an ORCA-Flash4.0 LT CMOS camera (Hamamatsu C11440-42 U).

### Stereology

To quantify TH-positive LC and SN neurons, the optical fractionator workflow (StereoInvestigator version 9, MicroBrightField Biosciences) was used. Therefore, tissue sections were stained against TH with DAB and counterstained with cresyl-violet as described above. To quantify LC cell numbers, five sections per animal containing the complete rostro-caudal extent of the LC region, separated by 120 μm, were selected. Contours including all TH-positive neurons of the LC were drawn, excluding neurons of the SubLC region. For quantification of TH-positive SN neurons, seven sections separated by 240 μm covering the complete caudo-rostral extent of the SN were used. Contours were drawn based on the cytoarchitectonic distribution of SN neurons [24] including SN pars compacta but excluding SN pars reticulata or ventral tegmental area neurons. Parameters used for counting were: grid size 100 × 100 μm, counting frame 85 × 85 μm, and 2 μm guard zones.

### Quantification of reactive micro- and astrogliosis

Triple immunofluorescence stainings were performed to visualize astro- and microgliosis using antibodies directed against GFAP for astroglia, IbA1 for microglia and TH to label LC neurons (Table 1). To quantify signs of reactive gliosis, we evaluated 5 LC sections of 6 animals per time point by measuring the optical density (OD) of the injected versus the non-injected side using FIJI. First, greyscale images were converted to 8 bit and the LC region was outlined with a rectangular contour (1200px x 800px). Then, OD was measured and lastly a background correction was performed by subtracting the mean background signal for every section. The background corrected OD values of all 5 sections of the injected side were summed and compared to the summed value of the non-injected side.

### Quantification of S129-phosphorylated aSYN

To analyze the degree of p-aSYN, a triple immunofluorescence staining against p-aSYN, human aSYN and TH was performed (Table 1). Five sections of 4 animals per time point, containing the complete rostro-caudal extent of the LC region, were selected for analysis. First, images were converted to 8 bit before making them binary. By using a preset intensity threshold, pixels were given either an intensity value of 255 (when positive for p-aSYN) or 0 (when negative for p-aSYN). The resulting p-aSYN signal intensity value was then divided by the area positive for non-phosphorylated aSYN. This ratio was calculated for all five sections and averaged per animal.

### Quantification of aSYN transport

Seven coronal sections (Bregma: + 4.28, + 2.86, + 1.18, + 0.38, − 0.58, − 3.16 and − 7.56) covering the complete mouse brain were stained against human aSYN (Syn 211) or Luc (Table 1) and the degree of aSYN accumulation was assessed by scoring human aSYN positive axons/cell bodies as follows: – no positive axons; + sparse (few positive axons); ++ mild (more positive axons); +++ moderate (many positive axons, covering almost the complete brain region) and ++++ severe pathology (large number of positive axons densely covering the complete brain region). (+) describes an intermediate state. Six animals per time point were analyzed and the scores for each brain region were averaged.

### Statistical analyses

In general, all data values are expressed as mean ± SEM or mean ± min/max. Differences were considered significant at *p* < 0.05. Multiple comparisons were made by one-way or two-way ANOVA analysis followed by Tukey’s or Sidak’s multiple comparisons test. To calculate correlations, Pearson’s correlation coefficient with 95% confidence interval was used. All statistical analyses were performed using GraphPad Prism version 7.00 (GraphPad Software, La Jolla California USA). Figures were created with Adobe Illustrator version 21.1 (Adobe Systems).

## Results

### rAAV vector-mediated overexpression of human A53T-aSYN in LC neurons

**Fig. 1:**
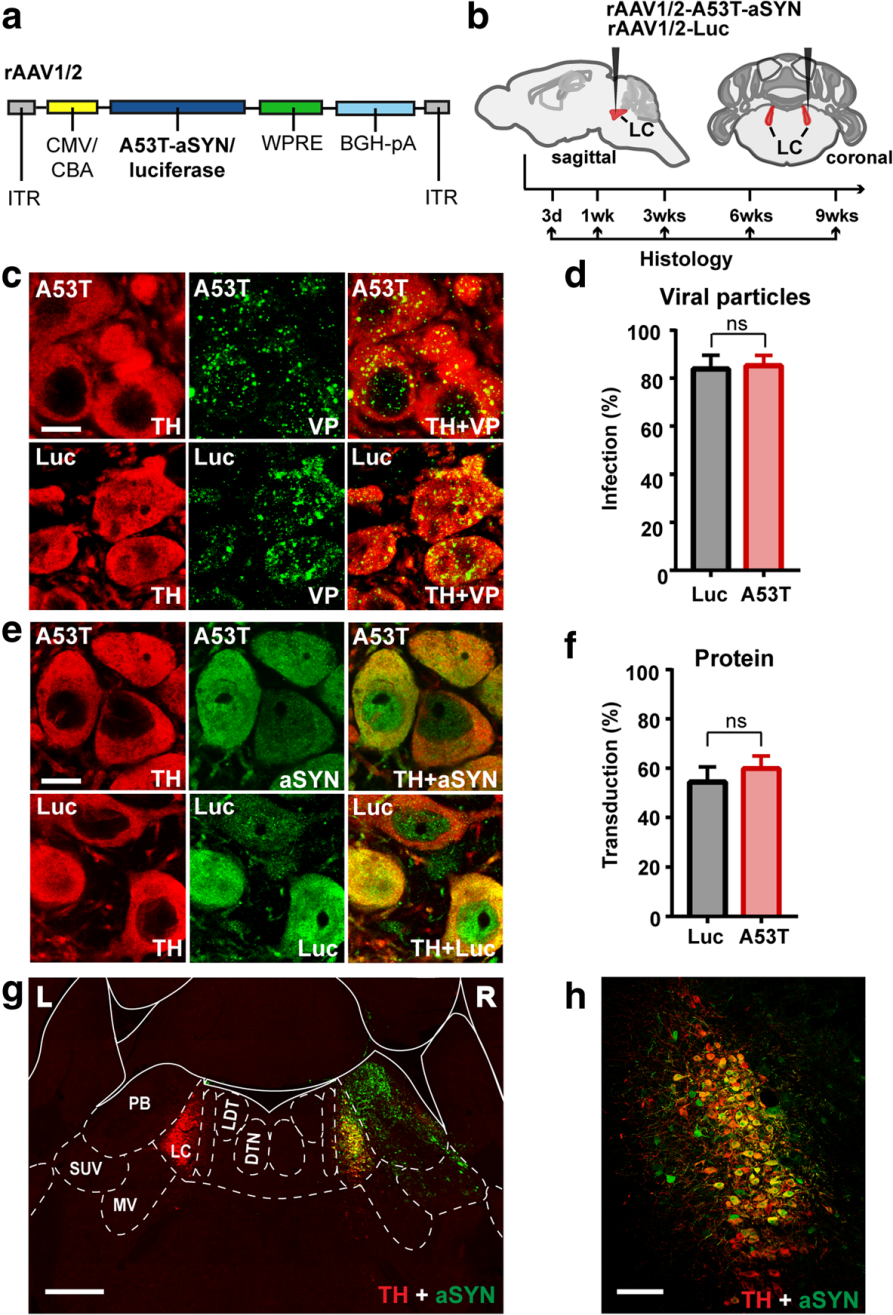
Locally induced protein overexpression via injection of rAAV vectors in the LC region. **a** rAAV1/2 vectors contain a chicken β-actin promoter hybridized with a CMV immediate early enhancer sequence (CMV/CBA) to drive expression of either A53T-aSYN or luciferase (control). ITR, inverted terminal repeat; WPRE, woodchuck hepatitis virus posttranscriptional regulatory element; BGH-pA, bovine growth hormone polyadenylation sequence. **b** Experimental design and schematic illustration of the injection site. Animals were consecutively sacrificed after 3 days, 1, 3, 6 and 9 weeks for immunohistochemical evaluation. **c**-**f** Analysis of the infection or transduction rates via double immunofluorescence staining for TH (red) and viral coating proteins (VP, green) (**c**, **d**) or TH (red) and human A53T-aSYN (green) or luciferase (green) (**e**, **f**), respectively. Co-localization of TH and VP indicates successful entry of viral particles, whereas co-localization of TH and A53T-aSYN/luciferase indicates successful protein expression. Student’s t-test revealed no significant difference between the transduction rates of the two vectors (*p* > 0.05, *n* = 3 animals per protein) (**d**, **f**). Values (mean ± SEM) represent the percentage (%) of TH-positive neurons that were also positive for VP, aSYN or Luc. **g** Overview of the pontine brainstem (Bregma: − 5.30 mm) stained against TH (red) and human aSYN (green) depicting the transduced area 3 days post-injection. Abbreviations: L, left; R, right; PB, parabrachial nucl.; SUV, superior vestibular nucl.; MV, medial vestibular nucl.; DTN, dorsal tegmental nucl.; LDT, laterodorsal tegmental nucl. **h** Higher magnification overview image of the TH-positive LC region (red) transduced with human A53T-aSYN (green). Scale bars 25 μm in **c**, **e**; 500 μm in **g** and 100 μm in **h**

To determine whether and in which time frame aSYN overexpression induces PD-like pathology in LC neurons we chose to overexpress human mutant A53T-aSYN by injecting rAAV1/2-A53T-aSYN [38, 44] unilaterally in the right LC region of wild-type mice (Fig. 1a, b). To verify that the resulting cellular effects were attributable to the aSYN protein itself, luciferase (Luc) was used as a control protein. To investigate time-dependent effects, animals were consecutively sacrificed after 3 days, 1, 3, 6 and 9 weeks (Fig. 1b).

By analyzing the first set of animals 3 days after viral injection, we confirmed that both vectors entered LC neurons equally (Fig. 1c, d), resulting in infection rates of 85.17 ± 2.53% for A53T-aSYN and 83.87 ± 3.31% for Luc (unpaired t-test, *p* = 0.77) (Fig. 1d). Double immunofluorescence stainings against TH and human aSYN or TH and Luc (Fig. 1e, f) revealed that both vectors induced protein expression already at this early time point with similar strength (A53T-aSYN 59.89 ± 2.95% and Luc 54.39 ± 3.57%, unpaired t-test, *p* = 0.30). Protein expression was mainly restricted to the LC covering the whole nucleus (Fig. 1g, h). In addition, a variable number of immuno-reactive cells were observed in the adjacent regions (ncl. Parabrachialis, Barrington’s nucleus, mesencephalic trigeminal nucleus and vestibular nuclei) (Fig. 1g). In LC neurons, cell bodies, as well as axons and dendrites were robustly labeled, indicating strong protein expression. Similar findings were observed for rAAV1/2-Luc injected animals. Notably, there was no aSYN or Luc signal in LC cells on the non-injected side at any time point (Fig. 1g). This allowed us to use the non-injected (left) side as an internal control.

### A53T-aSYN overexpression causes LC neurodegeneration

**Fig. 2:**
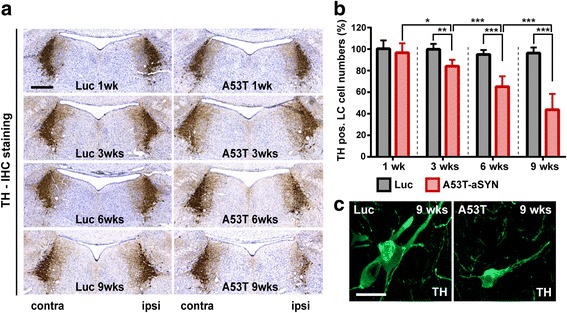
Progressive loss of TH-immunoreactive LC cells after rAAV1/2-A53T-aSYN injection. **a**, **b** Representative images (Bregma − 5.40 mm) and unbiased stereology of TH-positive LC-neurons in A53T-aSYN (*red bars, right column*) or Luc (*black bars, left column*) overexpressing animals. Values (mean ± SEM) are expressed as cell numbers on the injected side compared to non-injected side (%). *n* = 8 per time point and group, two-way ANOVA analysis followed by Tukey’s post-hoc test, *p < 0.05, **p < 0.01, ***p < 0.001. **c** Representative confocal images of neuronal morphology after 9 weeks of protein overexpression. Pyknotic cell bodies and dystrophic axons were observed in A53T-aSYN, but not in Luc overexpressing animals. Scale bars 250 μm in **a**, 50 μm in **c**

In the first set of experiments, the extent of aSYN induced LC cell loss was assessed with unbiased stereological quantification of TH-positive LC cells 1, 3, 6 and 9 weeks after viral vector delivery. In the A53T-aSYN group, significant degeneration of TH-positive LC cells was measured already 3 weeks post-injection, with 15.86 ± 2.09% cell loss compared to control side. Neurodegeneration increased progressively reaching 34.84 ± 3.39% after 6 weeks and 56.25 ± 5.19% after 9 weeks (Fig. 2a, b). Cell loss was homogenously distributed over the complete rostro-caudal extent of the LC. No cellular pathology was observed in the Luc control group at any investigated time point, confirming that neither the viral vector nor overexpression of a cytoplasmic protein was able to induce neurodegeneration in our model (Fig. 2a, b).

Moreover, immunofluorescent TH-stainings and subsequent confocal imaging revealed that A53T-aSYN, but not Luc overexpression was accompanied by qualitative changes of neuronal morphology, including dystrophic axons and pyknotic perikarya (Fig. 2c).

### Accumulation of phosphorylated-aSYN in the LC region

**Fig. 3:**
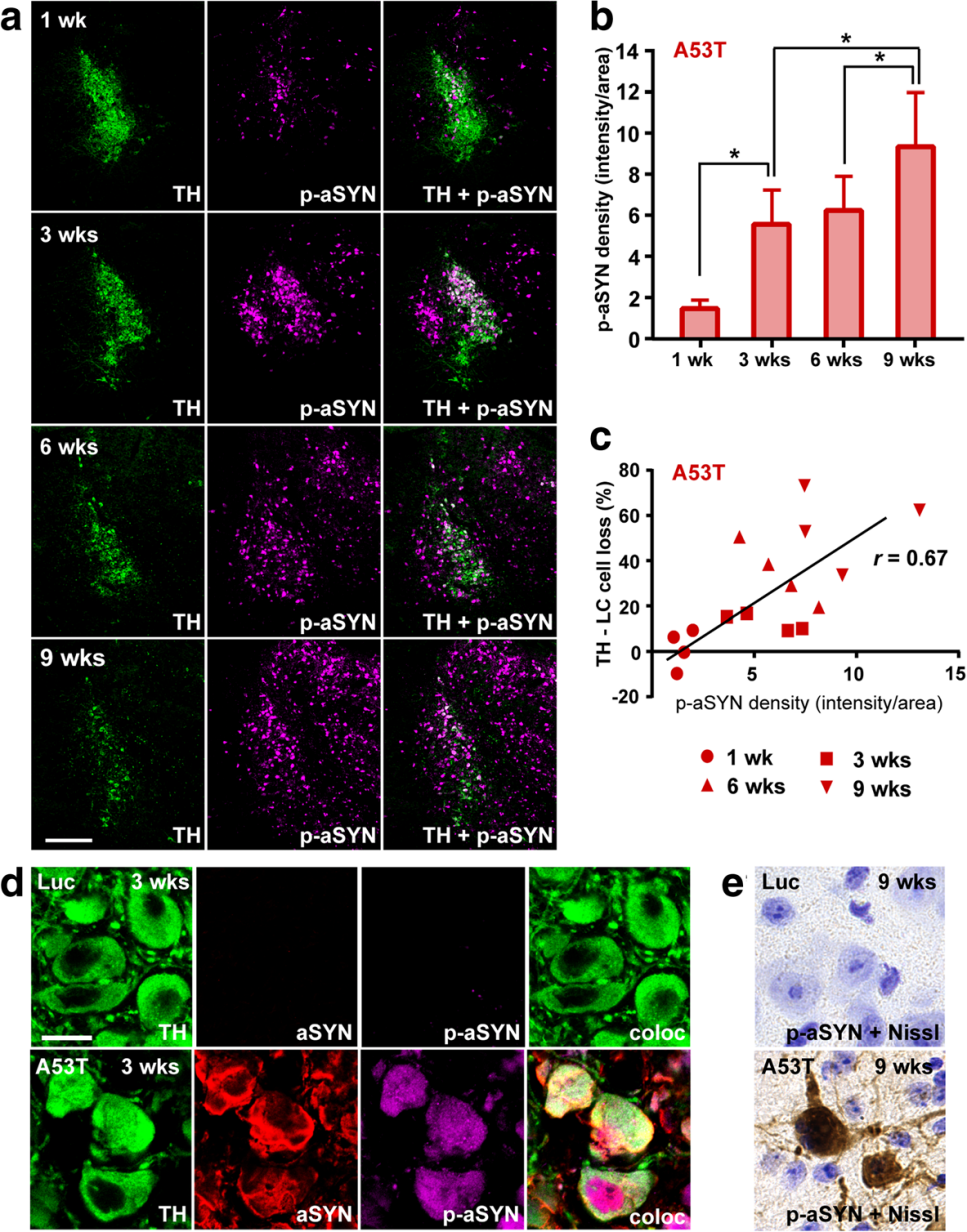
Progressive accumulation of phosphorylated aSYN (p-aSYN) in the LC region. **a**, **b** Representative images (Bregma − 5.40 mm) and quantification of p-aSYN in the LC region via double immunofluorescence staining for TH (green) and p-aSYN (magenta). Values are presented as mean ± SEM, *n* = 4 per time point and group, one-way ANOVA analysis followed by Tukey’s post-hoc test, *p < 0.05. **c** Robust correlation was observed between loss of TH-positive LC cells and accumulation of p-aSYN (*r* = 0.67, *p* < 0.05). **d** Confocal microscopy confirmed accumulation of p-aSYN (magenta) in TH-positive LC cells (green) 3 weeks post injection in A53T-aSYN (red) overexpressing animals (*lower row*). **e** p-aSYN immunoreactivity in the LC-region of A53T-aSYN overexpressing animals (*lower row*) 9 weeks post injection. No A53T-aSYN or p-aSYN immunoreactivity was observed in Luc overexpressing animals (*upper row*) at any time point. Scale bars 250 μm in **a**, 25 μm in **d**-**e**

Phosphorylation of aSYN at amino acid serine 129 (p-aSYN) is a commonly observed phenomenon in human PD brain tissue and in animal models artificially overexpressing aSYN [1, 25, 52, 72, 98]. In these models, the S129 phosphorylation is frequently used as an indicator for aSYN aggregation. In our current study, we measured the signal intensity of p-aSYN systematically via double immunofluorescence stainings for TH and p-aSYN. Our data revealed that A53T-aSYN overexpression led to strong and progressive phosphorylation of aSYN in LC neurons (Fig. 3a, b). Accumulation of p-aSYN started early with positive cells being observable already 1 week post-injection, reaching highest levels at the latest time point. Generally, the p-aSYN signal was homogenously distributed in the cytoplasm of TH-positive LC cells. In addition, robust labeling of the nucleus was observed (Fig. 3d, e). To exclude the possibility of non-specific antibody labeling we analyzed rAAV-Luc injected animals, which showed no signal for p-aSYN at any time point (Fig. 3d, e). Next, we wanted to quantify if the degree of phosphorylation correlated with the degree of noradrenergic cell loss. Therefore, the p-aSYN signal intensity values were plotted and correlated with the percentage of LC cell loss (Fig. 3c). The strong correlation (*r* = 0.67, *p* < 0.05) indicates that the degree of aSYN phosphorylation can be used as a predictor of aSYN toxicity in our model.

### Formation of proteinase K (PK)-resistant, p62-, Ubi-1- and aSYN-positive aggregates

**Fig. 4:**
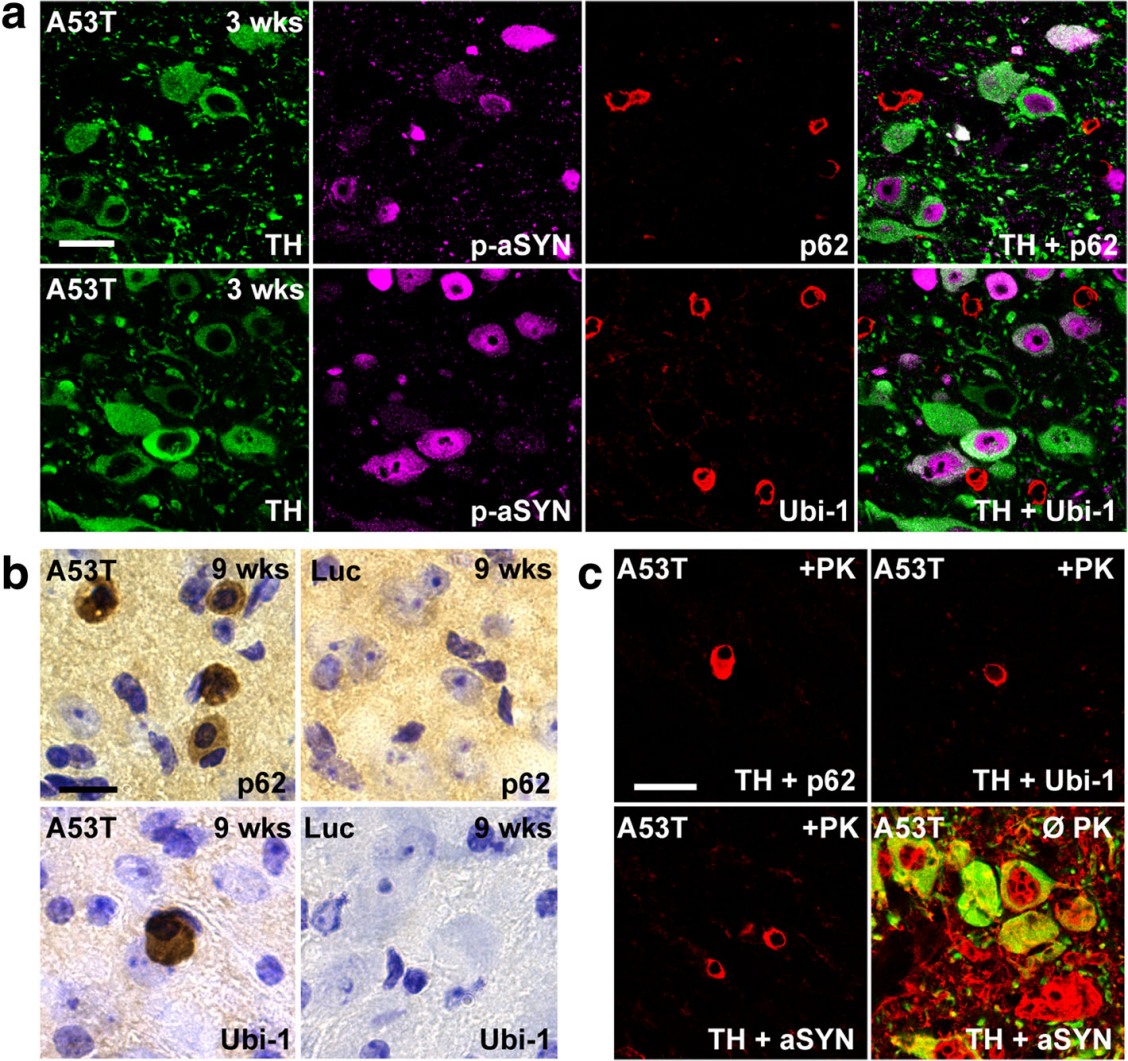
Formation of insoluble protein aggregates in A53T-aSYN overexpressing animals. **a** Staining for p62 (red, *upper row*) and Ubi-1 (red, *lower row*) revealed abundant p62- and Ubi-1-positive aggregates in the LC-region. These aggregates were found in close proximity to, but did not co-localize with TH-positive (green) LC cells, which were positive for p-aSYN (magenta). **b** Representative images of p62- (*upper left*) and Ubi-1 (*lower left*) positive aggregates in A53T-aSYN animals. No aggregates were observed in Luc overexpressing animals at any time point (*right column*). **c** p62-, Ubi-1 and aSYN stainings after proteinase K (PK) digestion or without digestion (Ø PK, *lower right*) confirmed that p62- (*upper left*), Ubi-1- (*upper right*) and human aSYN-positive (*lower left*) aggregates were insoluble. Scale bars 50 μm in **a** and **c**, 25 μm in **b**

**Fig. 5:**
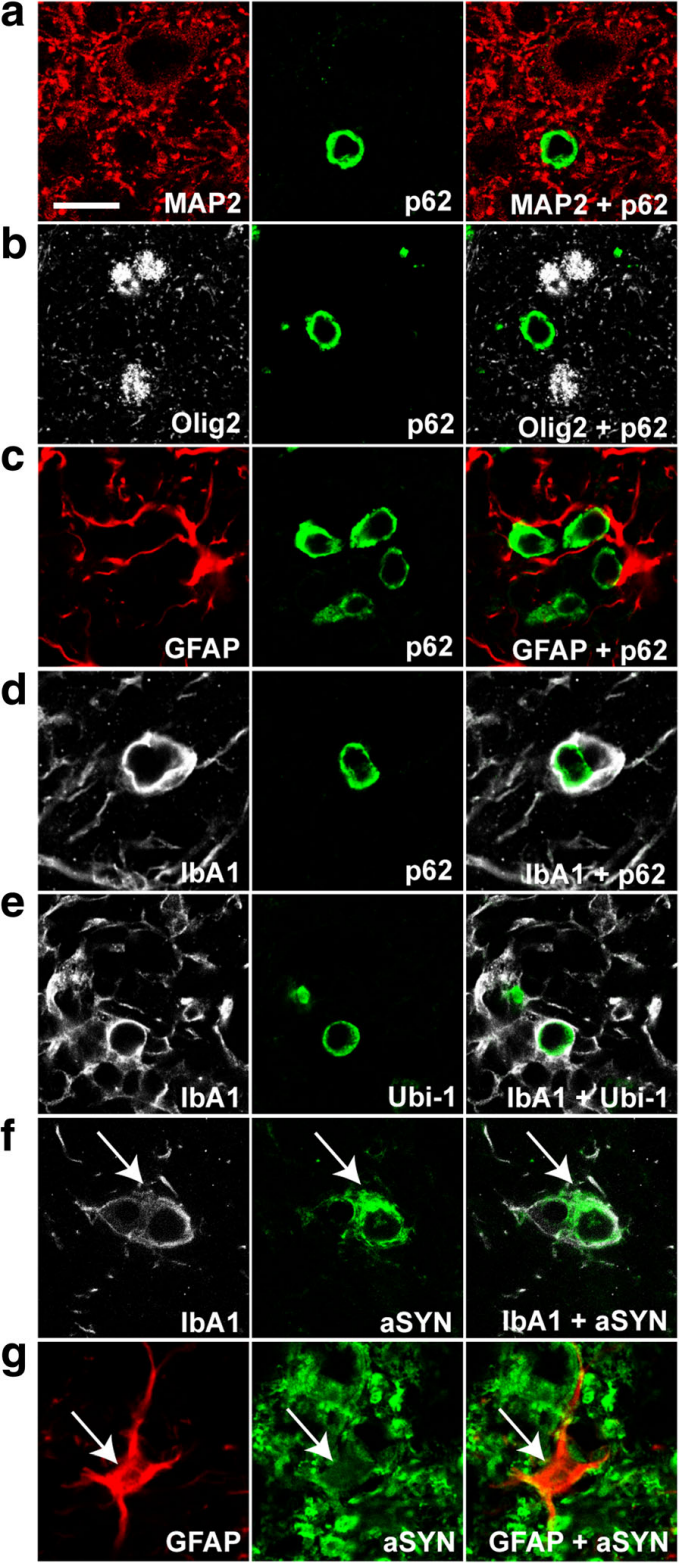
p62- and Ubi-1 positive aggregates co-localize with IbA1-positive microglia. **a**-**c** Representative confocal microscopy images of p62 (green, *second column*) and MAP2 (**a**, red, *first row*), Olig2 (**b**, gray, *second row*), or GFAP (**c**, red, *third row*) show no co-localization of p62 and the different cellular markers. In contrast, co-staining p62 (green) and IbA1 (**d**, gray, *fourth row*) or Ubi-1 (green) and IbA1 (**e**, gray, *fifth row*) revealed clear co-localization. IbA1 positive microglial cells (**f**, gray, *sixth row*) and GFAP-positive astroglia (**g**, red, *seventh row*) also co-stained for aSYN (*arrows* in **f**, **g**). Scale bar for **a**-**g** 25 μm

Lewy bodies in human PD brain tissue are characterized by immunoreactivity for insoluble (PK-resistant) aSYN, but also for a variety of other proteins, such as ubiquitin-1 (Ubi-1) and p62/SQSTM1/sequestosome-1 (p62) [33, 47]. Both of the latter proteins are implicated in the cellular clearance of aSYN. Occurrence of PK-resistant Ubi-1-positive aggregates indicates an overburdened proteasomal clearing system, while dysfunction of the lysosomal system can result in accumulation of p62-positive aggregates [73]. To test whether proteasomal and/or lysosomal clearance might be impaired in our model, we systematically screened A53T-aSYN and Luc overexpressing animals for p62- and Ubi-1-immunoreactivity. A53T-aSYN, but not Luc injected mice showed abundant p62- and Ubi-1-positive aggregates starting 3 weeks after viral vector delivery reaching highest numbers at the latest time point (Fig. 4a, b). Ubi-1-, as well as p62-positive inclusions appeared as small circular objects surrounding the nuclei of the cells (Fig. 4b) and were restricted to the ipsilateral side of injection. As in the previous experiments most of the p-aSYN signal was seen in TH-positive neurons (Fig. 3a, d), we expected a high rate of co-localization for p62 and Ubi-1 with the LC marker TH. However, the majority of p62 and Ubi-1 immunoreactivity was located next to TH-positive LC cells, suggesting that other cells are involved in this process (Fig. 4a). To elucidate in which cell type the p62-positive aggregates were located, double immunofluorescence stainings for p62 with MAP2 (neuronal marker), Olig2 (oligodendroglial marker), GFAP (astrocytic marker) or IbA1 (microglial marker) were performed. While p62 did not co-localize with MAP2 (Fig. 5a), Olig2 (Fig. 5b) or GFAP (Fig. 5c), we observed clear co-localization with IbA1 (Fig. 5d), indicating that the p62-positive inclusions were located in microglial cells. Moreover, we further confirmed that Ubi-1-positive aggregates were also located in microglia (Fig. 5e). Double immunofluorescence stainings for IbA1 and aSYN (Syn211) (Fig. 5f, arrow) and GFAP and aSYN (Syn211) (Fig. 5g, arrow) revealed that microglia, as well as astroglia exhibited human aSYN after 3 weeks of aSYN overexpression.

Next, we aimed to investigate if the observed p62- and Ubi-1-positive inclusions indeed consisted of insoluble aggregated proteins. Since PK resistance is accepted as a valid marker for the formation of insoluble aggregates in human PD samples and animal models [5, 38, 86], we digested tissue samples of A53T-aSYN and Luc injected mice of all time points with PK. As a result, numerous PK-resistant insoluble aggregates positive for p62, Ubi-1 and aSYN were found in A53T-aSYN injected mice (Fig. 4c). Notably, PK-resistant aSYN aggregates had the same shape and size as Ubi-1- and p62-inclusions. Further, all three kinds of aggregates started to appear 3 weeks after initiation of A53T-aSYN overexpression and were restricted to the site of viral injection. PK digestion and subsequent analysis of rAAV-Luc injected animals revealed no signal for aSYN, p62, Ubi-1 or Luc in any analyzed section.

### Targeted α-synucleinopathy induces reactive micro- and astrogliosis in the LC region

**Fig. 6:**
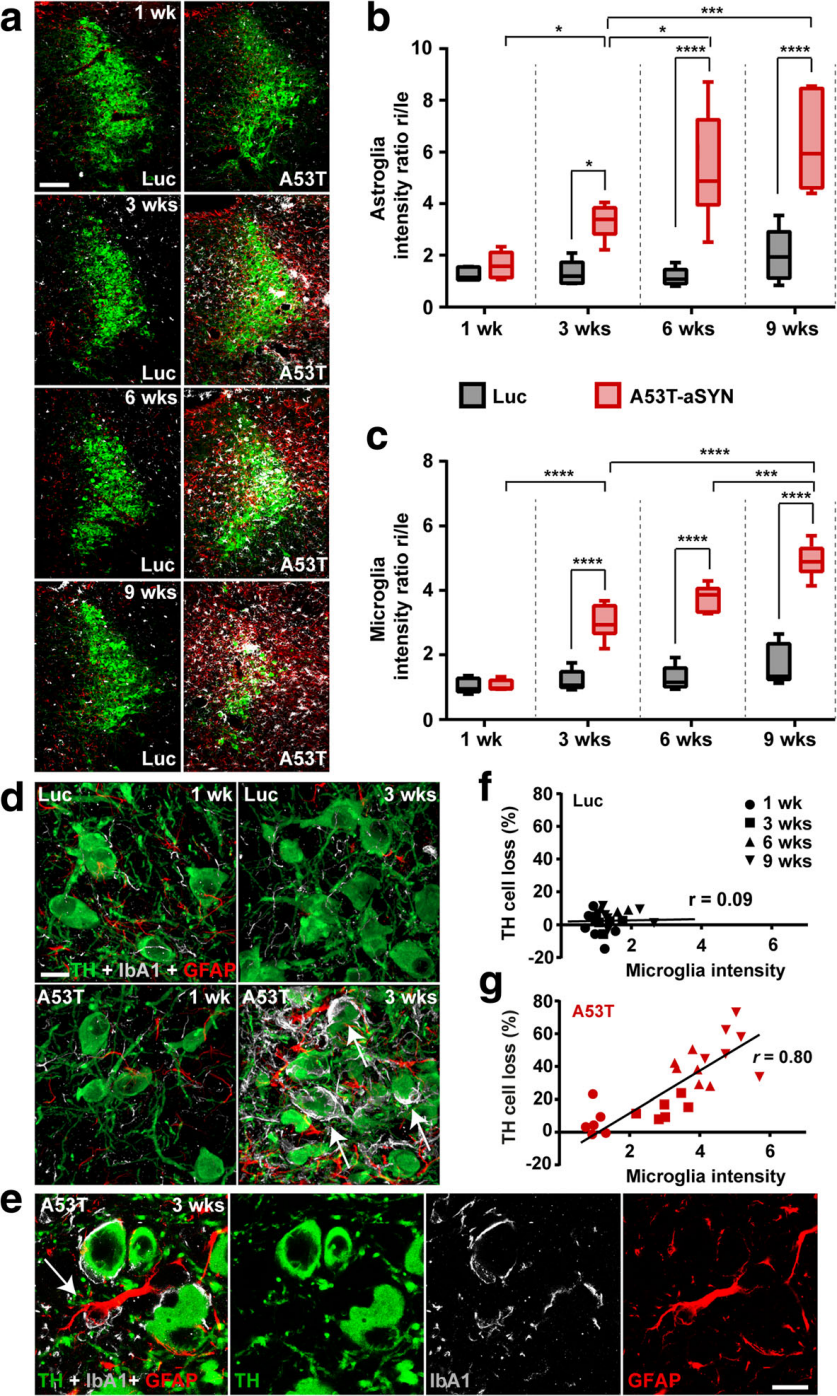
A53T-aSYN overexpression leads to a pronounced reactive micro- and astrogliosis in the LC-region. **a** Representative images of the LC region of Luc (*left column*) or A53T-aSYN (*right column*) injected animals stained for TH (green), IbA1 (gray) and GFAP (red) display a marked increase of micro- and astroglia over time in A53T-aSYN overexpressing mice. Quantification of GFAP (**b**) and IbA1 (**c**) signal intensity revealed a progressive increase of astro- and microglia signal in A53T-aSYN injected animals (*red boxes*) compared to Luc control (*black boxes*). Values (mean ± min/max) are expressed as the signal intensity ratio of the injected side compared to the non-injected side. *n* = 6 animals per time point and group. Two-way ANOVA analysis followed by Tukey’s post-hoc test, *p < 0.05, **p < 0.01, ***p < 0.001, ****p < 0.0001. **d** Reconstructed high magnification confocal images of the LC region showing physical contacts between TH-positive (green) LC cells and IbA1-positive micro- (gray) and GFAP-positive astroglia (red) after 3 weeks of A53T-aSYN overexpression (*lower right*). Engulfment (arrow) of TH-positive neurons by glial cells was only observed in A53T-aSYN expressing animals and not in Luc control mice (*upper row*). **e** Direct physical contacts were also observed between micro- and astroglia (arrow). **f**, **g** Correlating TH cell loss with the microglia intensity values indicates a strong association between increase of microglia and severity of TH cell loss in A53T-aSYN overexpressing animals (*r* = 0.80, *p* < 0.05), whereas there was no correlation in Luc expressing animals (*r* = 0.09, *p* > 0.05). Pearson’s correlation coefficient with 95% confidence interval. Scale bars 100 μm in **a**, 25 μm in **d** and **e**

Microglia activation and reactive astrocytes have been observed by respective PET imaging in human prodromal and manifest PD patients [26, 63], post-mortem PD brain samples [56, 62] and aSYN animal models [4, 87, 88]. Most of the studies using animal models focused on the impact of microglia activation following nigrostriatal degeneration. In the current study, we aimed to investigate whether a focally induced α-synucleinopathy in the LC region would lead to reactive micro- and astrogliosis. Therefore, a triple immunofluorescence staining for IbA1 (microglial marker), GFAP (astroglial marker) and TH was carried out and the intensity of fluorescence signal was quantified (Fig. 6a-c). Already 3 weeks of A53T-aSYN overexpression were sufficient to induce a 3.5-fold increase of astroglial signal intensity in the injected LC region compared to Luc control. The astrogliosis further progressed up to a 6-fold increase after 9 weeks (Fig. 6b). Simultaneously, a 3-fold signal increase for microglia was measured after 3 weeks of A53T-aSYN overexpression and a 5-fold increase after 9 weeks, compared to Luc (Fig. 6c). 3D reconstructed high magnification confocal images revealed a dense glial network in A53T-aSYN overexpressing animals, in which the remaining TH-positive LC neurons were embedded already 3 weeks after viral vector delivery (Fig. 6d). Abundant direct physical contacts between TH-positive LC neurons and astro- and microglia could be resolved. In addition, numerous LC cells appeared to be nearly completely engulfed by microglial processes (Fig. 6d, arrows). In contrast, overexpression of Luc did not lead to any significant increase of astro- or microglia intensity values (Fig. 6a-d). Besides the interaction of astro- and microglia with LC neurons, we also observed direct physical contacts between astrocytes and microglial cells (Fig. 6e, arrow).

To underline our hypothesis that the degree of aSYN-induced pathology is closely associated with the degree of microgliosis, we correlated the microglial intensity values with the percentage of LC neurodegeneration (Fig. 6f, g). This revealed a correlation coefficient of *r* = 0.80 (*p* < 0.05) for A53T-aSYN, whereas for the Luc overexpressing animals no significant correlation was found (*r* = 0.09, *p* > 0.05).

### Extensive transport of human A53T-aSYN to efferent brain regions

**Fig. 7:**
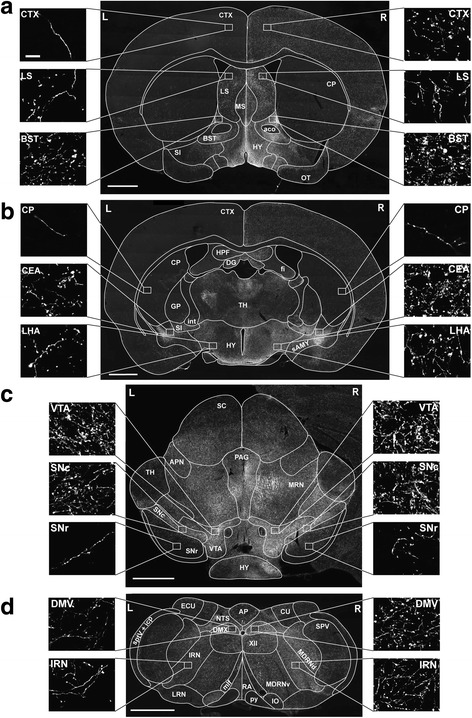
Widespread transport of human A53T-aSYN to interconnected brain regions. **a**-**d** Representative images of analyzed brain sections stained against human aSYN (Syn211) (Bregma + 0.50 mm **a**, Bregma − 0.94 mm **b**, Bregma − 3.16 mm **c**, Bregma − 7.56 mm **d**). *Scale bars* 1 mm in **a**-**d**, 25 μm in all high magnification images. Abbreviations: CTX, cortex; CP, caudoputamen; LS, lateral septal nucleus; MS, medial septal nucleus; aco, anterior commissure; BST, bed nuclei of stria terminalis; HY, hypothalamus; SI, substantia innominata; OT, olfactory tubercle; HPF, hippocampal formation; DG, dentate gyrus; fi, fimbria hippocampi; int, internal capsule; TH, thalamus; GP, globus pallidus; sAMY, striatum-like amygdalar nuclei; LHA, lateral hypothalamic area; CEA, central amygdalar nucleus; SC, superior colliculus; APN, anterior pretectal nucleus; PAG, periaqueductal gray; MRN, midbrain reticular nucleus; VTA, ventral tegmental area; SNc, substantia nigra pars compacta; SNr, substantia nigra pars reticulata; AP, area postrema; NTS, nucleus of the solitary tract; CU, cuneate nucleus; ECU, external cuneate nucleus; DMX, dorsal motor nucleus of the vagus nerve; XII, hypoglossal nucleus; SPV, spinal nucleus of the trigeminal; MDRNd, medullary reticular nucleus, dorsal part; MDRNv, medullary reticular nucleus, ventral part; IRN, intermediate reticular nucleus; IO, inferior olivary complex; py, pyramid; RA, raphe nuclei; mlf, medial longitudinal fascicle; LRN, lateral reticular nucleus; sptV, spinal tract of the trigeminal nerve; icp, inferior cerebellar peduncle; L, left (contralateral); R, right (ipsilateral)

**Fig. 8:**
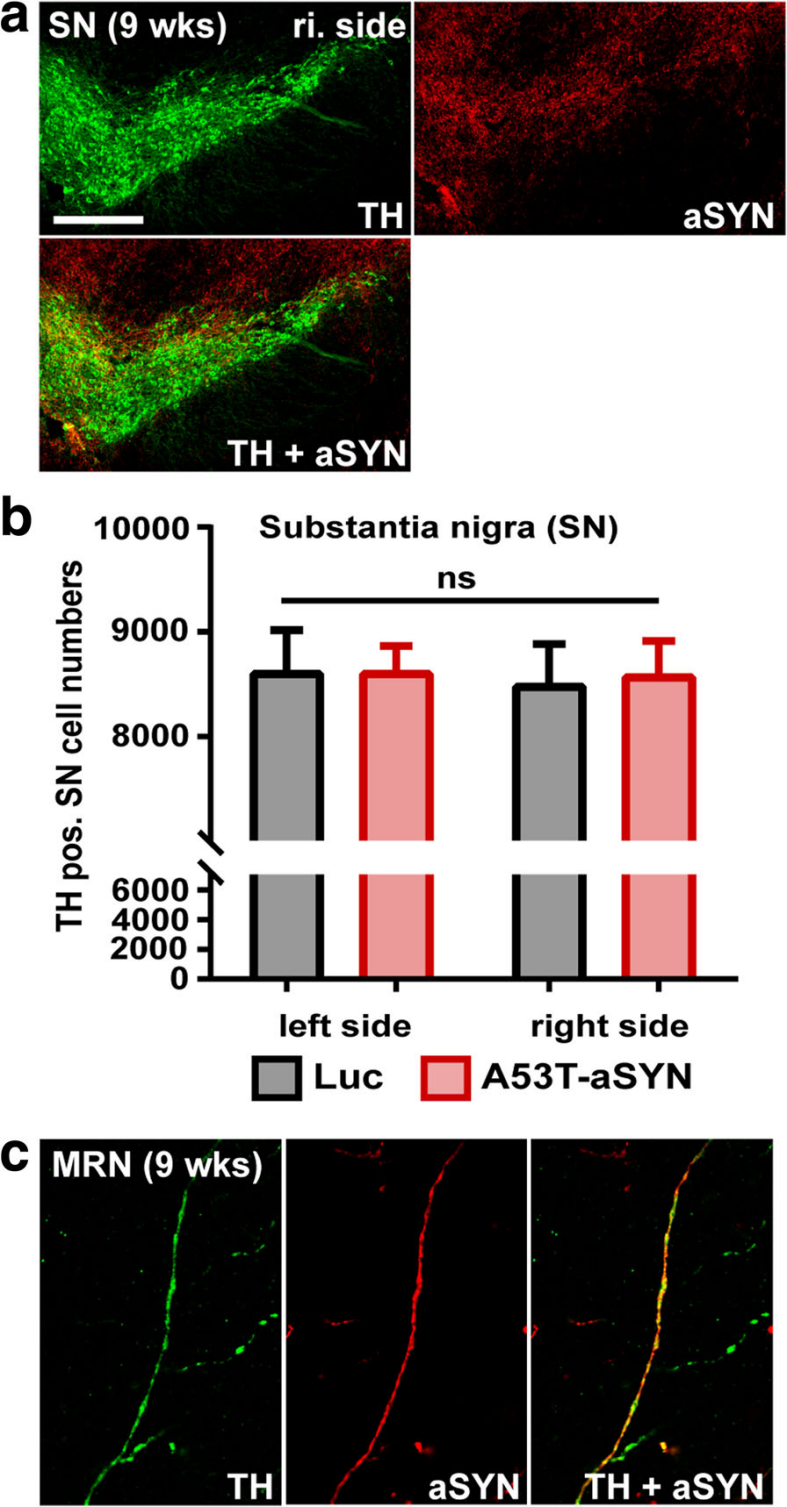
No SN cell loss after 9 weeks of A53T-aSYN overexpression in LC region. **a** Abundant human aSYN-positive (red, *upper right*) axons were observed in the TH-positive SN region (green, *upper left*) after 9 weeks of A53T-aSYN overexpression in the LC. In contrast, no aSYN-positive cell bodies could be detected. **b** Quantification of TH-positive SN neurons 9 weeks post injection revealed no significant difference between A53T-aSYN group (*red bars*) and Luc control group (*black bars*) for either side. Values are presented as mean ± SEM, *n* = 8 per group and side. One-way ANOVA analysis (*p* > 0.05). **c** Representative image of TH and human aSYN axonal co-localization in distant brain regions, exemplified for midbrain reticular nucleus (MRN). The majority of aSYN positive axons co-stained for TH indicating that they origin from the LC. Scale bars 250 μm in **a**, 25 μm in **c**

After investigating the local effects of A53T-aSYN overexpression, we addressed the question whether the aSYN pathology can propagate to anatomically connected brain regions. A variety of studies using overexpression of rAAV-aSYN or injection of preformed aSYN fibrils (PFF’s) have described transport or spread of aSYN to anatomically connected brain regions [35, 52, 54, 70, 77, 91]. To investigate the propagation of human A53T-aSYN after inducing the α-synucleinopathy in LC neuronal somata, we stained predetermined brain sections against human aSYN (Syn211) or Luc and rated the occurrence of aSYN- or Luc-positive axons or cell bodies (Table 2). While overexpression of Luc resulted in a staining pattern, which was limited to the injection site and absent in distant brain regions, we observed aSYN signal in a high number of brain regions in A53T-aSYN injected mice (Fig. 7). One week after injection of rAAV-A53T-aSYN in the right LC region, abundant aSYN-positive axons were observed in various brain regions which are known output regions of LC neurons [85]. The human aSYN signal was solely axonal and no aSYN-positive cell bodies were detected. Regions showing the strongest aSYN signal included the main olfactory bulb, lateral septal nucleus, diagonal band nucleus, bed nuclei of the stria terminalis, central amygdalar nucleus, periaqueductal gray, midbrain reticular nucleus, substantia nigra (SN) pars compacta and the ventral tegmental area (Table 2). We counted 36 brain regions, which contained human aSYN-positive axons after one week, indicating that human A53T-aSYN was transported rapidly along the axons towards the synaptic terminals in an anterograde direction. Despite the increase of axonal aSYN signal, no aSYN-positive cell bodies were detected outside of the LC region at any investigated time point, arguing against the hypothesis that human A53T-aSYN is released in LC output regions and taken up by synaptically connected cells in the short time frame of 9 weeks. This is highlighted by the finding that staining against p-aSYN revealed no signs of phosphorylation or aggregation of endogenous aSYN in distant brain regions after 9 weeks whereas the axons containing human (non-phosphorylated) A53T-aSYN stained positive for TH (Fig. 8c).

**Table 2:** Semiquantitative analysis of human aSYN-pathology in distant brain regions

		Injected (right) hemisphere Time of A53T-aSYN overexpression				Non-injected (left) hemisphere Time of A53T-aSYN overexpression			
		1 wk	3 wks	6 wks	9 wks	1 wk	3 wks	6 wks	9 wks
Bregma	Main olf. Bulb, gr. layer	+	++	++(+)	++	(+)	+	+	(+)
+4.28 mm	Inner plexiform layer	–	+(+)	+(+)	+	–	(+)	(+)	(+)
	Outer plexiform layer	(+)	+	+	+	–	(+)	(+)	(+)
Bregma	Cortex	+	++	++(+)	++	(+)	+	+	(+)
+ 2.96 mm	Main olf. Bulb	+	++	++(+)	++	(+)	(+)	+	(+)
	Ant. olf. Nucleus	+	+(+)	+(+)	+	–	(+)	(+)	(+)
	Lateral olf. Tract	(+)	+(+)	+	+	–	–	–	–
Bregma	Cortex	+	++(+)	++(+)	++(+)	–	+	+	+
+ 1.18 mm	Taenia tecta, dors. Part	+	++	++	+(+)	–	+	+	(+)
	Corpus callosum	(+)	+(+)	+	+	–	(+)	(+)	(+)
	Lateral septal nucleus	+(+)	++(+)	+++	++(+)	(+)	+	+(+)	+(+)
	Diagonal band nucleus	+(+)	++(+)	+++	++(+)	(+)	+	+(+)	+(+)
	Nucleus accumbens	+	+	+	+	–	(+)	(+)	(+)
	Caudoputamen	(+)	+	+	(+)	–	(+)	(+)	(+)
Bregma	Medial septal nucleus	+	+(+)	++	+(+)	(+)	+	+(+)	+
+ 0.38 mm	Cortex	(+)	++(+)	++(+)	++	–	+	+	+
	Bed ncl. of stria term.	++	+++	+++(+)	+++	(+)	+	++	++
	Magnocellular ncl.	(+)	+	++	++	–	(+)	+	+
	Hypothalamus	+	++(+)	++(+)	+++	–	+(+)	+(+)	++
	Lateral septal nucleus	(+)	++	++(+)	++(+)	–	+	+(+)	+(+)
	Caudoputamen	(+)	+	+	(+)	–	(+)	(+)	(+)
	Subst. innominata	+	+	+(+)	+(+)	(+)	(+)	+	+
Bregma	Central amygdalar ncl.	+	++	+++	+++(+)	(+)	+(+)	++	++
−0.58 mm	Subst. innominata	+(+)	++	++(+)	+++	+	+(+)	++	++
	Globus pallidus	(+)	+	+	+	–	(+)	(+)	(+)
	Hypothalamus	+	++	++(+)	++	(+)	+	+(+)	+(+)
	Lat. hypothalamic area	+(+)	++	+++	++(+)	(+)	+	++	++
	Fimbria	(+)	+	(+)	(+)	(+)	+	(+)	(+)
	Stria terminalis	+	+(+)	+(+)	+(+)	–	(+)	+	+
	Corpus callosum	–	+	+	+	–	–	(+)	(+)
	Cortex	(+)	++(+)	++(+)	++	–	+	+	+
Bregma	Cortex	–	+(+)	+(+)	+	–	(+)	(+)	(+)
−3.16 mm	Sup. colliculus, sens.	–	+(+)	+(+)	+	–	+	+	+
	Sup. colliculus, motor	(+)	+(+)	++	++	(+)	+(+)	++	++
	Periaqueductal gray	+(+)	++	++(+)	++(+)	(+)	+(+)	++	++
	Midbrain reticular ncl.	+(+)	++	++	++(+)	+	+(+)	++	++
	Red nucleus	(+)	+	+(+)	+	+	++	++(+)	++
	Ventral tegmental area	+	+(+)	++	++(+)	+(+)	++	++(+)	++(+)
	Subst. nigra, pars compacta	+(+)	+(+)	++	++	(+)	+	+(+)	+(+)
	Subst. nigra, pars reticulata	(+)	+	+	(+)	(+)	(+)	(+)	(+)
	Hippocampus	–	+	+(+)	+	–	(+)	+	+
	Interpeduncular ncl.	(+)	+(+)	++	++(+)	(+)	+(+)	++(+)	++(+)
	Thalamus	(+)	+(+)	++	++	(+)	+	+(+)	+(+)
Bregma	Dorsal motor ncl. of n. X	–	+	+(+)	+(+)	–	(+)	+	+
−7.56 mm	Hypoglossal ncl.	–	+	+	+	(+)	(+)	+	(+)
	Parvicell. reticular ncl.	+(+)	+(+)	+(+)	+(+)	(+)	(+)	+	+
	Intermediate ret. ncl.	+	+	++	+(+)	–	(+)	+	+
	Medullary reticular ncl.	+	+(+)	+(+)	+(+)	–	+	+	+
	Lateral ret. ncl.	–	(+)	+	(+)	–	(+)	(+)	(+)
	Ncl. of the solitary tract	–	(+)	+	+(+)	–	(+)	+	+
	Spinal ncl. of the n. V	–	(+)	(+)	(+)	–	–	(+)	(+)
	Area postrema	(+)	(+)	+	+	(+)	(+)	+	+
	Arbor vitae	–	+	+	+	–	+	+	+
	Gr. layer (cerebellum)	+	+(+)	+(+)	+(+)	(+)	+	+	+
	Mol. layer (cerebellum)	–	+	+	+	–	+	+	+

Occurrence of human aSYN-positive axons was graded out of seven coronal brain sections as follows: – no positive axons; + sparse (few positive axons); ++ mild (more positive axons); +++ moderate (many positive axons, covering almost the complete brain region) and ++++ severe pathology (large number of positive axons densely covering the complete brain region); (+) describes an intermediate state between two categories to allow a more accurate description. *n* = 6 per time point. Abundance of aSYN-positive axons increased over time and was more prominent in the injected (right) hemisphere. The signal for human aSYN was solely axonal and no aSYN-positive cell bodies were detected

### No substantia nigra (SN) cell loss after 9 weeks of human A53T-aSYN overexpression in LC neurons

Already after 1 week of A53T-aSYN overexpression in LC neurons, human aSYN positive axons passing by DA SN neurons could be detected. After 9 weeks, SN neurons were densely surrounded by aSYN containing axons (Fig. 8a) but no human aSYN signal was observed in the somata of SN cells. Stereological quantification of TH-positive SN neurons (Fig. 8b) revealed no significant difference of TH-immunoreactive neurons between A53T-aSYN compared to Luc overexpressing mice neither for the left nor for the right SN (One-way ANOVA; *p* > 0.05). This result points out that LC degeneration, in combination with profound local axonal aSYN accumulation was not sufficient to induce degeneration of DA SN neurons within the relatively short period of 9 weeks.

## Discussion

Degeneration of the LC noradrenergic system is a key event during PD pathogenesis in the prodromal phase of the disease. In this study, we present the first targeted LC α-synucleinopathy mouse model, which replicated cardinal features of human PD pathology. We have designed our rAAV vector-based overexpression model to generate robust and rapid induction of aSYN pathology, including phosphorylation and aggregation of aSYN, noradrenergic neurodegeneration, development of dystrophic axon morphology, signs of proteasomal and lysosomal dysfunction and prominent neuron-glial interactions. Furthermore, the herein characterized aSYN transport pattern allows investigating the effects of aSYN-induced LC neurodegeneration on anatomically connected LC output structures.

### Progressive S129 phosphorylation and formation of PK-resistant aSYN-positive aggregates

Phosphorylation of aSYN at amino acid S129 is a dominant pathological modification of aSYN [1] since approximately 90% of aSYN in human Lewy bodies is phosphorylated at this position, whereas only 4% of soluble aSYN exhibits this posttranslational modification [25]. In PD animal models, phosphorylation at S129 is used as a key marker to investigate an induced α-synucleinopathy and its occurrence has often been interpreted as formation of aSYN aggregates [10, 52, 65, 72, 87]. In our current study, we show abundant and over time increasing S129-phosphorylation of aSYN in the cytoplasm and nucleus of LC cells (Fig. 3a, d). Previous studies have pointed out that aSYN has different cellular localizations. Beside the presynaptic and cytoplasmic localization, a nuclear occurrence of aSYN is known [80]. Nuclear p-aSYN has been observed in previous studies where aSYN was overexpressed [30, 96] and it could be shown that nuclear aSYN interacts with histone molecules. It was even suggested that the S129-phosphorylation may play an important role for the nuclear translocation of aSYN [64]. To confirm that phosphorylation of aSYN was accompanied by formation of high molecular weight aSYN aggregates we performed PK digestion experiments that revealed small circular aSYN positive inclusion bodies restricted to the injection site (Fig. 4c). Our model thereby reproduces a key feature of the LC pathology observed in human PD patients. Importantly, since PK digestion led to the destruction of all soluble proteins it did not allow us to investigate if the developing aSYN-positive inclusions are located in neurons or glial cells. The observed discrepancy between a high amount of p-aSYN-positive cells and a relatively limited number of aSYN-positive PK-resistant inclusions raises the question whether aSYN S129-phosphorylation can solely be used as a sufficient marker for aSYN aggregation. Our data indicate that S129-phosphorylation is an important indicator for aSYN pathology, but immunohistochemistry for other aggregation markers should be added to confirm the occurrence of aSYN aggregates [69, 90].

### Formation of p62- and Ubi-1-positive proteinaceous inclusions in microglia

Additional markers, which are also commonly accepted to investigate protein aggregation and simultaneously serve as indicators for dysfunction of the proteasomal or lysosomal protein degradation system include Ubi-1 and p62 [33, 47]. Based on previous reports, which showed close co-localization of p-aSYN and Ubi-1 or p62 [54, 74], we expected to find overlap of these markers in our model. But notably, all p62 and Ubi-1 aggregates were located next to p-aSYN positive LC cells (Fig. 4a). Co-staining p62 and Ubi-1 with different glial and neuronal markers revealed that the p62 and Ubi-1 inclusions were located in IbA1-positive microglial cells (Fig. 5d, e). Further, we also show that microglia exhibited human aSYN, probably as a result of local aSYN uptake or phagocytosis of aSYN containing cellular debris. (Fig. 5f). The possibility that the microglial cells were transduced by rAAV1/2-A53T seems to be unlikely, since the used rAAV1/2 vector possesses a high neuronal tropism [44] and triple stainings for IbA1 (microglia), GFAP (astroglia) and aSYN revealed no aSYN-immunoreactivity 3 days after viral vector delivery within micro- or astroglial cells. Despite this, we cannot completely exclude the possibility of microglia transduction by the initial injection of rAAV1/2-A53T. Deposition of internalized aSYN aggregates in microglia has already been observed in vitro [49], but our study represents (to our knowledge) the first in vivo evidence of inclusion formation in microglial cells. We hypothesize that these p62- und Ubi-1-positive aggregates develop de novo in microglial cells possibly because of massive human aSYN uptake, which exceeds the lysosomal degradation capacities and leads to protein aggregation.

Currently we can only speculate why p62 and Ubi-1 reactivity was observed in microglia but absent in LC neurons. Previous studies have shown that p62-positive inclusions co-localize with Ubi-1 not only in neuronal but also in glial cells in neurodegenerative diseases including Alzheimer’s disease, dementia with Lewy bodies and PD [47]. Furthermore, it has been shown that microglia rapidly internalize aSYN thereby representing the most efficient scavengers of neuronal released aSYN [48, 71]. By clearing aSYN, microglia might actively delay accumulation of aSYN and maturation of aSYN aggregates in LC neurons. One could hypothesize that in our model aSYN is rapidly released from LC neurons and taken up by microglia, which in turn leads to microglial but not neuronal accumulation of p62 und Ubi-1 aggregates. A longer duration of the experiment may clarify the question, whether Ubi-1- and p62-positive inclusions might also develop in LC neurons.

### Reactive astro- and microglia and their implication in aSYN-induced LC pathology

Another key aspect in several animal models in which aSYN was injected or overexpressed [87, 88, 100] is the profound involvement of reactive astro- and microgliosis during the development of the aSYN pathology. It has been shown that activated microglial cells, besides their implication in clearing aSYN, are able to trigger the release of inflammatory cytokines and accelerate the production of reactive oxygen species, thereby likely contributing to the process of neurodegeneration [32, 42, 100]. In our model, LC cells were surrounded by a massive network of astro- and microglia already after 3 weeks of aSYN overexpression, with many microglial cells almost completely engulfing the surviving LC neurons (Fig. 6d, arrows). This early induction of microgliosis (Fig. 6c) is in line with previous findings where microgliosis even preceded the onset of neurodegeneration [4, 13]. Furthermore, we observed a strong correlation between the increase of microglial signal and LC cell loss (Fig. 6g), implying the conclusion that reactive microglial cells are important modulators of aSYN-induced toxicity not only in the dopaminergic SN but also in the noradrenergic LC. Microgliosis was accompanied by severe and progressively increasing astrogliosis. Reactive astrocytes surrounded and partially engulfed LC neurons. Furthermore, they formed direct physical contacts with reactive microglia (Fig. 6e, arrow) and exhibited clear signal for human aSYN (Fig. 5g). Importantly, reactive astrocytes can also take part in clearing aSYN by endocytosis and degradation in their lysosomal system [50, 76]. Furthermore, they interact closely with microglia and release pro- and anti-inflammatory molecules [18, 57]. Our LC model exemplifies this close interdependency between neurons, micro- and astroglia. We show that glial cells are highly involved in the process of aSYN degradation and that glial dysfunction or failure could be a factor of PD progression. However, it should also be considered that the LC itself plays a central role in decreasing neuroinflammation [20]. Noradrenaline is able to suppress the expression of pro-inflammatory cytokines in glial cells while simultaneously elevating the expression of anti-inflammatory markers [19, 59]. Hence, it is reasonable to assume that loss of LC neurons additionally increases the neuroinflammatory response and contributes to the progressive increase of micro- and astroglial activity seen in our model.

### Anterograde axonal transport of aSYN to LC output regions

To slow or prevent the progression of PD, it is essential to investigate if and how the α-synucleinopathy propagates within the brain. Recent evidence [36, 45, 52, 54, 70] suggests that toxic aSYN species formed in a small number of cells can spread trans-synaptically to distant but anatomically connected brain regions where they act as seeds to trigger the formation of insoluble aSYN aggregates [29, 54]. Furthermore, cell culture experiments have demonstrated that aSYN can be taken up by cells and transported in both the retrograde and anterograde direction [15, 23, 94]. The noradrenergic LC has a broad input-output connectome [82, 85] making this brain region suitable to investigate trans-neuronal spread. Moreover, Iba and colleagues [37] have demonstrated in a tauopathy model that injections of synthetic tau fibrils were able to induce tau pathology in LC neurons which then propagated to LC afferents and efferents. To investigate if this also translates into our LC aSYN overexpression model we systematically analyzed and scored the aSYN pathology after 1, 3, 6 and 9 weeks (Fig. 7, Table 2). Our results indicate that the overexpressed human A53T-aSYN, once produced in the cytoplasm of LC neurons, is only transported in the anterograde direction towards the synaptic terminals, as abundant aSYN-positive axons and terminals in efferent LC regions co-stained for TH (Fig. 7, Fig. 8c, Table 2). The broad LC output connectome [85] likely explains this high amount of aSYN-positive axons in distant brain regions of the ipsilateral but also contralateral hemisphere. The mild aSYN pathology of the contralateral (non-injected) side can be explained by LC projections crossing the midline and innervating brain structures of the contralateral hemisphere [39]. In contrast to the profound axonal aSYN immunoreactivity, we found no aSYN-positive cell bodies outside of the LC region, arguing against trans-neuronal spread of aSYN in the relatively short time frame of 9 weeks. The absence of Luc-positive axons in LC output regions might be explained on one hand by protein size (Luc 62 kDa vs. aSYN 15 kDa) and on the other hand by the naturally presynaptic localization of aSYN [7, 89]. We therefore conclude that the aggregation prone aSYN species created by overexpression of human A53T-aSYN in the LC region are not transferred to other neuronal populations within the investigated 9 weeks. A longer time period and subsequently higher aSYN burden in the LC system may be necessary to enable such a transfer at later time points. This is in line with the finding that despite the severe degree of axonal aSYN accumulation in the SN region after 9 weeks of A53T-aSYN overexpression in the LC region, no statistically significant SN neurodegeneration was observed (Fig. 8).

### Open questions and limitations

In this study, we have decided to overexpress human mutant A53T-aSYN by injection of a previously well-established rAAV vector. The vector used in this study has proven effective in inducing progressive neurodegeneration of SN neurons by several groups in several PD animal models [28, 34, 38, 43, 44]. In this context, it would be of relevance to investigate whether overexpression of wild-type aSYN in the LC would have led to a different histopathological phenotype. Considering the lower rate of β-sheet and fibril formation of wild-type aSYN compared to the A53T-aSYN variant [14, 51], one could hypothesize that overexpression of wild-type aSYN might lead to milder histopathological alterations. However, this has to be demonstrated in the LC model in a further study. For this initial study we have focused on a relatively short time frame of up to 9 weeks which allowed us to characterize the initial, local and time-dependent histopathological alterations of LC neurons caused by A53T-aSYN. Since 9 weeks is likely too short to observe the full neuropathology, a future study containing longer observation times of up to 52 weeks or even longer would be suggested. This would allow to further investigate whether trans-synaptic spread of aSYN and subsequent degeneration of dopaminergic SN neurons occur at a later time-point. As our study primarily aims to address the histopathological consequences of A53T-aSYN overexpression in LC neurons, we have not carried out a behavioral assessment. Nevertheless, the model would benefit from a thoroughly carried out behavioral characterization, including sleep recordings covering the possible occurrence of any non-motor or subtle motor symptoms.

## Conclusions

In a time, in which on one hand the clinical research focus shifts away from the neurodegeneration of the dopaminergic nigrostriatal pathway towards the prodromal stages of PD and on the other hand the first potentially disease modifying therapies enter clinical testing [61], animal models mimicking the prodromal phase of PD are needed. In this study, we have reproduced cardinal histopathological features of the human LC PD-pathology, delineated the time-course of noradrenergic neurodegeneration and characterized robust histological markers, which are sufficient to assess the pathological changes in a quantitative and qualitative way. Taken together, this animal model may contribute to the research on the pathophysiology of the prodromal stage of PD. Further studies with longer observation times and additional characterization (e.g. behavioral assessment, biochemical analyses) are required to determine whether the herein presented model will prove helpful in the development and testing of disease-modifying therapy.

## Abbreviations

aSYN: α-synuclein
DA: Dopaminergic
LC: Locus coeruleus
Luc: Luciferase
p-aSYN: Phosphorylated α-synuclein
PB: Phosphate buffer
PBS: Phosphate buffered saline
PD: Parkinson’s disease
PFA: Paraformaldehyde
PK: Proteinase K
rAAV: Recombinant adeno-associated viral
RBD: REM sleep behavior disorder
SN: Substantia nigra
TH: Tyrosine hydroxylase
